# Singular-Value-Decomposition-Based Matrix Surgery

**DOI:** 10.3390/e26080701

**Published:** 2024-08-17

**Authors:** Jehan Ghafuri, Sabah Jassim

**Affiliations:** School of Computing, The University of Buckingham, Buckingham MK18 1EG, UK; sabah.jassim@buckingham.ac.uk

**Keywords:** condition number, singular value decomposition, topological data analysis, SVD surgery

## Abstract

This paper is motivated by the need to stabilise the impact of deep learning (DL) training for medical image analysis on the conditioning of convolution filters in relation to model overfitting and robustness. We present a simple strategy to reduce square matrix condition numbers and investigate its effect on the spatial distributions of point clouds of well- and ill-conditioned matrices. For a square matrix, the SVD surgery strategy works by: (1) computing its singular value decomposition (SVD), (2) changing a few of the smaller singular values relative to the largest one, and (3) reconstructing the matrix by reverse SVD. Applying SVD surgery on CNN convolution filters during training acts as spectral regularisation of the DL model without requiring the learning of extra parameters. The fact that the further away a matrix is from the non-invertible matrices, the higher its condition number is suggests that the spatial distributions of square matrices and those of their inverses are correlated to their condition number distributions. We shall examine this assertion empirically by showing that applying various versions of SVD surgery on point clouds of matrices leads to bringing their persistent diagrams (PDs) closer to the matrices of the point clouds of their inverses.

## 1. Introduction

Despite the remarkable success and advancements of deep learning (DL) models in computer vision tasks, there are serious obstacles to the deployment of AI in different domains related to the challenge of developing deep neural networks that are both robust and generalise well beyond the training data [1]. Accurate and stable numerical algorithms play a significant role in creating robust and reliable computational models [2]. The source of numerical instability in DL models is partially due to the use of a large number of parameters/hyperparameters and data that suffer from floating-point errors and inaccurate results. In the case of convolutional neural networks (CNNs), an obvious contributor to the instability of their large volume of weights is the repeated action of backpropagation algorithms for controlling the growth of the gradient descent to fit the model’s performance to the different patches of training samples. This paper is concerned with empirical estimation of CNN training-caused fluctuations in the condition numbers of various weight matrices as a potential source of instability at convolutional layers and their negative effects on overall model performance. We shall propose a spectral-based approach to reduce and control the undesirable fluctuation.

The *condition number* κ(A) of a square n×n matrix *A*, which is considered as a linear transformation $R^{n\times n}\to R$, measures the sensitivity of computing its action to perturbations to input data and round-off errors, which are defined as sup∥Ax∥/∥x∥ over the set of nonzero *x*. The condition number depends on how much the calculation of its inverse suffers from underflow (i.e., how much det(A) is significantly different from 0). Stable action of A means that small changes in the input data are expected to lead to small changes in the output data, and these changes are bound by the reciprocal of the condition number. Hence, the higher the condition number of A is, the more unstable A’s action is in response to small data perturbations, and such matrices are said to be ill-conditioned. Indeed, the distribution of the condition numbers of a random matrix simply describes the loss in precision, in terms of the number of digits, as well as the speed of convergence due to ill-conditioning when solving linear systems of equations iteratively [3]. Originally, the condition number of a matrix was introduced by A. Turing in [4]. Afterwards, the condition numbers of matrices and numerical problems were comprehensively investigated in [5,6,7]. The most common efficient and stable way of computing κ(A) is by computing the SVD of *A* and calculating the ratio of *A*’s largest singular value to its smallest non-zero one [8].

J. W. Demmel, in [6], investigated the upper and lower bounds of the probability distribution of condition numbers of random matrices and showed that the sets of ill-posed problems including matrix inversions, eigenproblems, and polynomial zero finding all have a common algebraic and geometric structure. In particular, Demmel showed that in the case of matrix inversion, the further away a matrix is from the set of noninvertible matrices, the smaller is its condition number. Accordingly, the spatial distributions of random matrices in their domains are indicators of the distributions of their condition numbers. These results provide clear evidence of the viability of our approach to exploit the tools of topological data analysis (TDA) to investigate the condition number stability of point clouds of random matrices. In general, TDA can be used to capture information about complex topological and geometric structures of point clouds in metric spaces with or without prior knowledge about the data (see [9] for more detail). Since the early 2000s, applied topology has entered a new era exploiting the persistent homology (PH) tool to investigate the global and local shape of high-dimensional datasets. Various vectorisations of persistence diagrams (PDs) generated by the PH tool encode information about both the local geometry and global topology of the clouds of convolution filters of CNN models [10]. Here, we shall attempt to determine the impact of the SVD surgery procedure on the PDs of point clouds of CNNs’ well- and ill-conditioned convolution filters.

**Contribution:** We introduce a singular-value-decomposition-based matrix surgery (SVD surgery) technique to modify the matrix condition numbers that is suitable for stabilising the actions of ill-conditioned convolution filters in point clouds of image datasets. The various versions of our SVD surgery preserve the norm of the input matrix while reducing the norm of its inverse away from non-invertible matrices. PH analyses of point clouds of matrices (and those of their inverses) post SVD surgery bring the PDs of point clouds of filters of convolution filters and those of their inverses closer to each other.

## 2. Background to the Motivating Challenge

The ultimate motivation for this paper is related to specific requirements that arose in our challenging investigations of how to “train an efficient slim convolutional neural network model capable of learning discriminating features of Ultrasound Images (US) or any radiological images for supporting clinical diagnostic decisions”. In particular, the developed model’s predictions are required to be robust against tolerable data perturbation and less prone to overfitting effects when tested on unseen data.

In machine learning and deep learning, vanishing or exploding gradients and poor convergence are generally due to an ill-conditioning problem. The most common approaches to overcome ill-conditioning are regularisation, data normalisation, re-parameterisation, standardisation, and random dropouts. When training a deep CNN with extremely large datasets of “natural” images, the convolution filter weights/entries are randomly initialised, and the entries are changed through an extensive training procedure using many image batches over a number of epochs, at the end of each of which, the back-propagation procedure updates the filter entries for improved performance. The frequent updates of filter entries result in non-negligible to significant fluctuation and instability of their condition numbers, causing sensitivity of the trained CNN models [11,12]. CNN model sensitivity is manifested by overfitting, reduced robustness against noise, and vulnerability to adversarial attacks [13].

Transfer learning is a common approach when developing CNN models for the analysis of US (or other radiological) image datasets, wherein the pretrained filters and other model weights of an existing CNN model (trained on natural images) are used as initialising parameters for retraining. However, condition number instabilities increase in the transfer learning mode when used for small datasets of non-natural images, resulting in suboptimal performance and the model suffering from overfitting.

## 3. Related Work

Deep learning CNN models involve a variety of parameters, the complexity of which are dominated by the entries of sets of convolution filters at various convolution layers as well as those of the fully connected neural network layers. The norms and/or variances of these parameters are the main factors considered when designing initialisation strategies to speed up training optimisation and improve model performance in machine and deep learning tasks. Currently, most popular CNN architectures initialise these weights using zero-mean Gaussian distributions with controlled layer dependent/independent variances. Krizhevsky et al. [14] use a constant standard deviation of 0.01 to initialise the weights in each layer. Due to the exponentially vanishing/growing gradient and for compatibility with activation functions, *Glorot* [15], or *He* [16], weights are initialised with controllable variances per layer. For Glorot, the initialised variances depend on the number of in/out neurons, while He initialisation of the variances is closely linked to their proposed parameterised rectified activation unit (PReLU), which is designed to improve model fitting with little overfitting risk. In all these initialisation strategies, no explicit consideration is given to the filters’ condition numbers or their stability during training. In these cases, our investigations found that, post training, almost all convolution filters are highly ill-conditioned, and hence, this adversely affects their use in transfer learning for non-natural images. More recent attempts to control the norm of the network layer were proposed in GradInit [17] and MetaInit [18]. These methods can accelerate the convergence while improving model performance and stability. However, both approaches require extra trainable parameters, and controlling the condition number during training is not guaranteed.

Recently, many research works have investigated issues closely related to our objectives by imposing orthogonality conditions on trainable DL model weights. These include orthonormal and orthogonal weight initialisation techniques [19,20,21], orthogonal convolution [22], orthogonal regularisers [23], orthogonal deep neural networks [24], and orthogonal weight normalisation [25]. Recalling that orthogonal/orthonormal matrices are optimally well conditioned, these publications indirectly support our hypothesis on the link between DL overfitting and condition numbers of learnt convolution filters. Although the instability of weight matrices’ condition numbers are not discussed explicitly, these related works fit into the emerging paradigm of spectral regularisation of NN layer weight matrices. For example, J. Wang et al. [22] assert that imposing orthogonality on convolutional filters is ideal for overcoming training instability of DCNN models and improves performance. Furthermore, A. Sinha [23] point out that an ill-conditioned learnt weight matrix contributes to a neural network’s susceptibility to adversarial attacks. In fact, their orthogonal regularisation aims to keep the learnt weight matrix’s condition number sufficiently low, and they demonstrate its increased adversarial accuracy when tested on the natural image datasets of MNIST and F-MNIST. S. Li et al. in [24] note that existing spectral regularisation schemes are mostly motivated to improve training for empirical applications and conduct a theoretical analysis of such methods using bounds on the concept of generalisation error (GE) measures that are defined in terms of the training algorithms and the isometry of the application feature space. They conclude that the optimal bound of the GE is attained when each weight matrix of a DNN has a spectrum of equal singular values, and they call such models OrthDNNs. To overcome the high computation requirements of strict OrthDNNs, they define approximate OrthDNNs by periodically applying their singular value bounding (SVB) scheme of hard regularisation. In general, controlling weights’ behaviours during training has proven to accelerate the training process and reduce the likelihood of overfitting the model to the training set, e.g., weight standardisation [26], weight normalisation/reparameterization [27], centred weight normalisation [28], and using Newton’s iteration controllable orthogonalization [29]. Most of the these proposed techniques have been developed specifically to deal with trainable DL models for the analysis of natural images, and one may assume that these techniques are used frequently during training after each epoch/batch. However, none of the known state-of-the-arts DL models seem to implicitly incorporate these techniques. In fact, our investigations of these commonly used DL models revealed that the final convolution filters are highly ill-conditioned [11].

Our literature review revealed that reconditioning and regularisation have long been used in analytical applications to reduce/control the ill-conditioning computations noted. In the late 1980s, E. Rothwell and B. Drachman [30] proposed an iterative method to reduce the condition number in ill-conditioned matrix problem that is based on regularising the non-zero singular values of the matrix. At each iteration, each diagonal entry in the SVD of the matrix is appended with a ratio of a regularising parameter to the singular value. This algorithm is not efficient enough to be used for our motivating challenge. In addition, the change of the norm is dependent on the regularising parameter.

In recent years, there has been a growing interest in using TDA to analyse point clouds of various types and complexities of datasets. For example, significant advances and insights have been made in capturing local and global topological and geometric features in high-dimensional datasets using PH tools, including conventional methods [31]. TDA has also been deployed to interpret deep learning and CNN learning parameters at various layers [11,32,33] and to integrate topology-based methods in deep learning [34,35,36,37,38,39]. We shall use TDA to assess the spatial distributions of point clouds of matrices/filters (and their inverses) before and after SVD surgery for well- and ill-conditioned random matrices.

## 4. Topological Data Analysis

In this section, we briefly introduce persistent homology preliminaries and describe the point cloud settings of randomly generated matrices to investigate their topological behaviours.

**Persistent homology of point clouds:** Persistent homology is a computational tool of TDA that encapsulates the spatial distribution of point clouds of data records sampled from metric spaces by recording the topological features of a gradually triangulated shape by connecting pairs of data points according to an increasing distance/similarity sequence of thresholds. For a point cloud *X* and a list ${\left\{\alpha_{i}\right\}}_{0}^{m}$ of increasing thresholds, the shape S(X) generated by this TDA process is a sequence ${\left\{S{\left(X\right)}_{i}\right\}}_{0}^{m}$ of simplicial complexes ordered by inclusion. The Vietoris–Rips simplicial complex (VR) is the most commonly used approach to construct S(X) due to its simplicity, and Ripser [40] is used to construct VR. The sequence of distance thresholds is referred to as a *filtration* of S(X). The topological features of S(X) consist of the number of holes or voids of different dimensions, which is known as the *Bettie* number, in each constituents of ${\left\{S{\left(X\right)}_{i}\right\}}_{0}^{m}$. For j≤0, the *j*-th Bettie number $B_{j}\left(S{\left(X\right)}_{i}\right)$ is obtained, respectively, by counting $B_{0}$ = #(connected components), $B_{1}$ = #(empty loops with more than three edges), $B_{2}$ = #(3D cavities bounded by more than four faces), etc. Note that $B_{j}\left(S_{i}\left(X\right)\right)$ is the set of generators of the *j*-th singular homology of the simplicial complex $S_{i}\left(X\right)$. The TDA analysis of *X* with respect to a filtration ${\left\{\alpha_{i}\right\}}_{0}^{m}$ is based on the persistency of each element of $B_{j}\left(S{\left(X\right)}_{i}\right)$ as i→m. Here, the persistency of each element is defined as the difference between its birth (first appearance) and its death (disappearance). It is customary to visibly represent $B_{j}\left(S{\left(X\right)}_{i}\right)$ as a vertically stacked set of barcodes, with each element having a horizontal straight line joining its birth to its death. For more-detailed and rigorous descriptions, see [41,42,43]). For simplicity, the barcode set and the PD of the $B_{j}\left(S_{i}\left(X\right)\right)$ are referred to by $H_{j}$.

Analysis of the resulting PH barcodes of point clouds in any dimension is provided by the **persistence diagram** (PD) formed by a multi-set of points in the first quadrants of the plane (x=birth, y=death) above or on the line y=x. Each marked point in the PD corresponds to a generator of the persistent homology group of the given dimension and is represented by a pair of coordinates (birth,death). To illustrate these visual representations of PH information, we created a point cloud of 1500 points sampled randomly on the surface of a torus:

$$T=\{\left(x,y,z\right)\in R^{3}:{\left(\sqrt{x^{2}+y^{2}}-a\right)}^{2}+z^{2}=b^{2}\}$$

**Figure 1:**
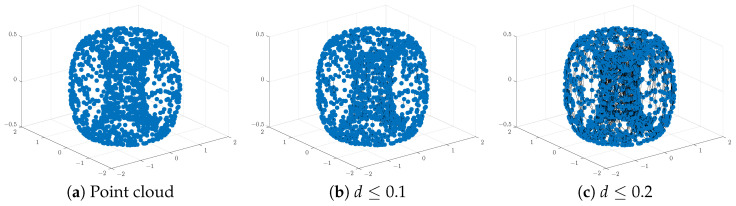
An illustration of a point cloud: (**a**) points from a toru, (**b**,**c**) connecting nearby points up to the distances *d* = 0.1 and 0.2, respectively.

**Figure 2:**
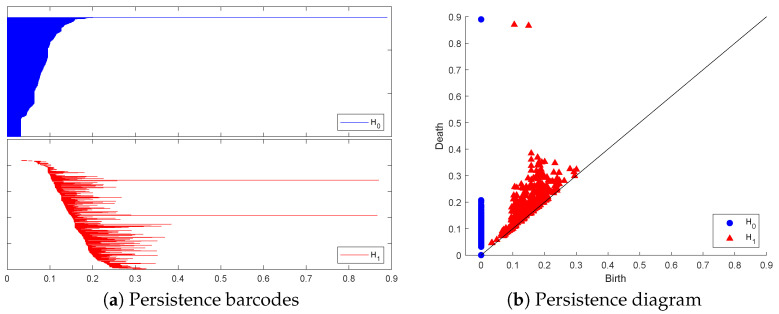
The topological representation of the torus point cloud as persistence barcodes and diagram.

Figure 1 and Figure 2 below display this point cloud together with the barcodes and PD representation of its PH in both dimensions. The two long 1−dim persisting barcodes represent the two empty discs whose Cartesian product generates the torus. The persistency lengths of these two holes depend on the radii (a−b,b) of the generating circles. In this case, a=2b. The persistency lengths of the set of shorter barcodes are inversely related to the point cloud size. Noisy sampling will only have an effect on the shorter barcodes.

Demmel’s general assertion that the further away a matrix is from the set of non-invertible matrices, the smaller is its condition number [6] implies that the distribution of condition numbers of a point cloud of filters is linked to its topological profile as well as that of the point cloud of their inverses. In relation to our motivating application, the more ill-conditioned the convolutional filter is, the closer it is to being non-invertible, resulting in unstable feature learning. Accordingly, the success of condition-number-reducing matrix surgery can be indirectly inferred by its ability to reduce the differences between the topological profiles (expressed by PDs) of point clouds of filters and those of their inverses. We shall first compare the PDs of point clouds of well-conditioned matrices and ill-conditioned ones, and we do the same for the PDs of their respective inverse point clouds.

Determining the topological profiles of point clouds using visual assessments of the corresponding point clouds’ persistent barcodes/diagrams is subjective and cumbersome. A more quantitatively informative way of interpreting the visual display of PBs and PDs can be obtained by constructing histograms of barcode persistency records in terms of uniform binning of birth data. Bottleneck and Wasserstein distances provide an easy quantitative comparison approach but may not fully explain the differences between the structures of PDs of different point clouds. In recent years, several feature vectorisations of PDs have been proposed that can be used to formulate numerical measures to distinguish topological profiles of different point clouds. The easiest scheme to interpret is the statistical vectorisation of persistent barcode modules [44]. Whenever reasonable, we shall complement the visual display of PDs with an appropriate barcode binning histogram of barcodes’ persistency, alongside computing the bottleneck and Wasserstein distances using the GUDHI library [45] to compare the topological profiles of point clouds of matrices.

**Figure 3:**
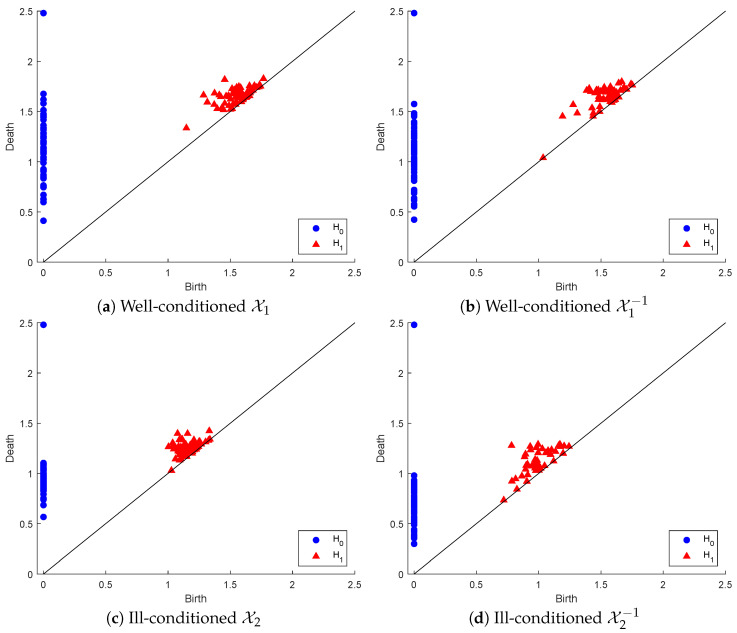
Persistence diagrams of point clouds representing well-conditioned and ill-conditioned matrices and their inverses.

To illustrate the above process, we generated a set of $10^{4}$ random Gaussian filters of size 3 × 3 matrices sorted in ascending order of their condition number, and we created two point clouds: (1) $X_{1}$ of the 64 matrices with the lowest condition numbers and (2) $X_{2}$ with the 64 matrices of the highest condition numbers. $X_{1}$ is well-conditioned, with condition numbers in the range [1.19376, 1.67], while $X_{2}$ is highly ill-conditioned, with condition numbers in the range [621.3677, 10,256.2265]. Below, we display the PDs in both dimensions of $X_{1}$, $X_{2}$ and their inverse point clouds in Figure 3.

In dimension zero, there are marginal differences between the connected component persistency of $X_{1}$ and that of $X_{1}^{-1}$. In contrast, considerable differences can be found between the persistence of the connected components of $X_{2}$ and that of $X_{2}^{-1}$. In dimension one, there are slightly more marginal differences between the hole persistency of $X_{1}$ and that of $X_{1}^{-1}$. However, these differences are considerably more visible between the hole persistency of $X_{2}$ and that of $X_{2}^{-1}$. One easy observation in both inverse point clouds, as opposed to the original ones, is the early appearance of a hole that dies almost immediately, being very near to the line death = birth.

A more informative comparison between the various PDs can be discerned by examining Table 1 below, which displays the persistency-death-based binning of the various PDs. Note that in all cases, there are 64 connected components born at time 0. The pattern and timing of death (i.e., merging) of connected components in the well-conditioned point clouds $X_{1}$ and $X_{1}^{-1}$ are nearly similar; however, in the case of ill-conditioned point clouds, most connected components of $X_{2}^{-1}$ merge much earlier than those of $X_{2}$.

The above results are analogous to Demmel’s result in that the well-conditioned point cloud exhibits similar topological profiles to that of its inverse point cloud, while the topological profile of the ill-conditioned point cloud differs significantly from that of its inverse. In order to estimate the proximity of the PDs of the well- and ill-conditioned point clouds to those of their inverses, we computed both the bottleneck and the Wasserstein. The results are included in Table 2 below, which also includes these distances between other pairs of PDs. Again, both distance functions confirm the close proximity of the PD of $X_{1}$ with that of $X_{1}^{-1}$ in comparison to the significantly bigger distances between the PDs of $X_{2}$ and $X_{2}^{-1}$.

Next, we introduce our matrix surgery strategy and the effects of various implementations on point clouds of matrices, with emphasis on the relations between the PDs of the output matrices and those of their inverse point clouds.

## 5. Matrix Surgery

In this section, we describe the research framework to perform matrix surgery that aims to reduce and control the condition numbers of matrices. Suppose matrix $A\in R^{m\times n}$ is non-singular and is based on a random Gaussian or uniform distribution. The condition number of A is defined as:

(1) $$\kappa \left(A\right)=∥A∥∥A^{-1}∥$$

where ∥.∥ is the norm of the matrix. In this investigation, we focus on the Euclidean norm ($L_{2}$-norm), where κ(A) can be expressed as:

$$\kappa \left(A\right)=\sigma_{1}/\sigma_{n}$$

where $\sigma_{1}$ and $\sigma_{n}$ are the largest and smallest singular values of *A*, respectively. A matrix is said to be ill-conditioned if any small change in the input results in big changes in the output, and it is said to be well-conditioned if any small change in the input results in a relatively small change in the output. Alternatively, a matrix with a low condition number (close to one) is said to be well-conditioned, while a matrix with a high condition number is said to be ill-conditioned, and the ideal condition number of an orthogonal matrix is one. Next, we describe our simple approach of modifying singular-value-matrix-based SVD since the condition number is defined by the largest and smallest singular values. We recall that the singular value decomposition of a square matrix $A\in R^{n\times n}$ is defined by:

(2) $$A=U\Sigma V^{T}$$

where $U\in R^{m\times m}$ and $V\in R^{n\times n}$ are left and right orthogonal singular vectors (unitary matrices); diagonal matrix $\Sigma =diag\left(\sigma_{1},\ldots ,\sigma_{n}\right)\in R^{m\times n}$ are singular values, where $\Sigma =\sigma_{1}\geq \sigma_{2}\geq ...\geq \sigma_{n}\geq 0$. SVD surgery, described below, is equally applicable to rectangular matrices.

### 5.1. SVD-Based Surgery

In the wide context, SVD surgery refers to the process of transforming matrices to improve their conditioning numbers. In particular, it targets matrices that are far from having orthogonality/orthonormality characteristics to replace them with improved well-conditioned matrices by deploying their left and right orthogonal singular vectors along with the new singular value diagonal matrix. SVD surgery can be realised in a variety of ways according to the expected properties of the output matrices to fit the use case. Given any matrix *A*, SVD surgery on *A* outputs a new matrix of the same size as follows: 
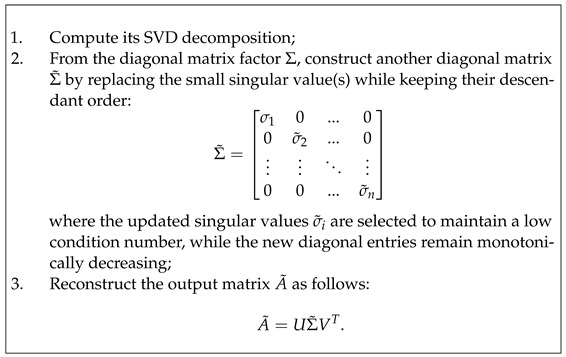


Changes to the singular values amount to rescaling the effect of the matrix action along the left and right orthogonal vectors of *U* and *V*, and the monotonicity requirement ensures reasonable control of the various rescalings. The orthogonal regularisation scheme of [22] and the SVB scheme of [24] do reduce the condition numbers when applied for improved control of overfitting of DL models trained on natural images, but both make changes to all the singular values and cannot guarantee success for the application of DL training of US image datasets. Furthermore, the SVB scheme is a rather strict form of SVD-based matrix surgery for controlling the condition numbers, but no analysis is conducted on the norms of these matrices or their inverses.

Our strategy for using SVD surgery is specifically designed for the motivating application and aims to reduce extremely high condition number values, preserve the norm of the input filters, and reduce the norm of their inverses away from non-invertible ones. Replacing all diagonal singular value entries with the largest singular value will produce an orthogonal matrix with a condition number equal to one, but this approach ignores or reduces the effect of significant variations in the training data along some of the singular vectors, leading to less effective learning. Instead, we propose a less drastic, application-dependent strategy for altering singular values. In general, our approach involves scaling all singular values to be less than $<\sigma_{1}$ in order to minimise $\sigma_{1}-\sigma_{n}$ while ensuring the maintenance of their monotonicity property. To reduce the condition numbers of an ill-conditioned matrix, it may only be necessary to adjust the relatively low singular values to bring them closer to $\sigma_{1}$. There are numerous methods for implementing such strategies, including the following linear combination scheme. Here, we follow a less drastic strategy to change singular values:
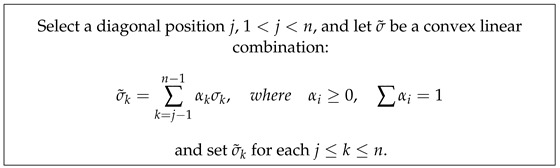


The value of *j* can be chosen to be any singular value that is very close to $\sigma_{1}$, and the linear combination parameters can be customised based on the application and can possibly be determined empirically. In extreme cases, this strategy allows for the possibility of setting $\sigma_{k}=\sigma_{j}$ for all k>j. This is rather timid in comparison to the orthogonal regularisation strategies, which preserve the monotonicity of the singular values. Regarding our motivating application, parameter choices would vary depending on the layer, but the linear combination parameters should not significantly rescale the training dataset features along the singular vectors. While SVD surgery can be applied to inverse matrices, employing the same replacement strategy and reconstruction may not necessarily result in a significant reduction in the condition number.

**Example:** Suppose *B* is a square matrix with n=3 that is drawn from a normal distribution with mean μ=0 and standard deviation σ=0.01 as follows:

$$B=\left[\begin{array}{ccc} -0.01960899999 & 0.02908008031 & -0.01058180258\\ -0.00197698226 & 0.00825218894 & -0.00468615581\\ -0.01207845485 & 0.01378971978 & -0.00272469409 \end{array}\right]$$

Singular values of *B* are $\Sigma =diag(\sigma_{1},\sigma_{2},\sigma_{3})$, and it is possible to modify and reconstruct ${\tilde{B}}_{1}$, ${\tilde{B}}_{2}$, and ${\tilde{B}}_{3}$ by replacing one and/or two singular values such that ${\tilde{\Sigma }}_{1}=diag\left(\sigma_{1},\sigma_{2},\sigma_{2}\right)$, ${\tilde{\Sigma }}_{2}=diag\left(\sigma_{1},{\tilde{\sigma }}_{2},{\tilde{\sigma }}_{3}\right)$, and ${\tilde{\Sigma }}_{3}=diag\left(\sigma_{1},\sigma_{1},\sigma_{1}\right)$, respectively. New singular values in ${\tilde{\Sigma }}_{2}$ are convex linear combinations such that ${\tilde{\sigma }}_{2}=2\sigma_{1}/3+\sigma_{2}/3$ and ${\tilde{\sigma }}_{3}={\tilde{\sigma }}_{2}$. After reconstruction, the condition numbers of ${\tilde{B}}_{1}$, ${\tilde{B}}_{2}$, and ${\tilde{B}}_{3}$ are significantly lower compared to those of the original matrix, as shown in Table 3, by using the Euclidean norm.

### 5.2. Effects of SVD Surgery on Large Datasets of Convolution Filters and Their Inverses

**Figure 4:**
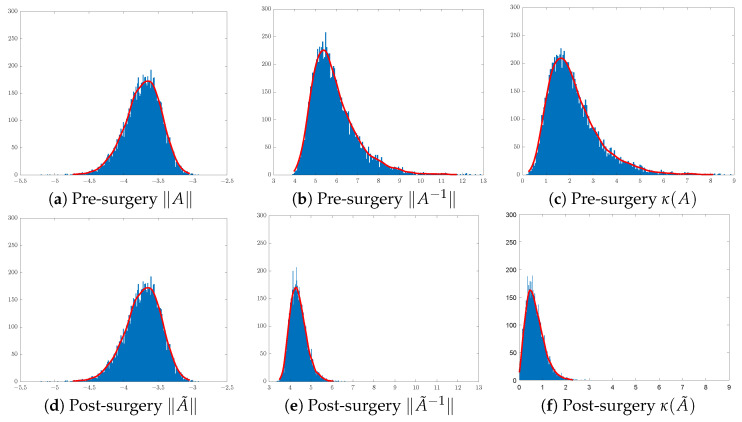
Distribution of 3×3 matrices pre- and post-surgery: (**a**,**d**) original matrix norms, (**b**,**e**) inverse matrix norms, and (**c**,**f**) matrix condition numbers.

**Figure 5:**
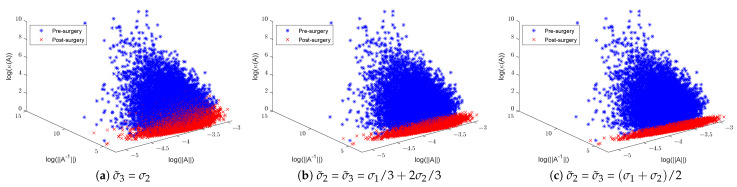
Illustration of 3×3 random Gaussian matrices pre- and post-matrix surgery, displaying norms, inverse norms, and logarithmic condition numbers: (**a**) $\sigma_{3}$ replaced with $\sigma_{2}$ and (**b**,**c**) $\sigma_{2}$ and $\sigma_{3}$ replaced with a new linear combination of $\sigma_{1}$ and $\sigma_{2}$.

In training CNN models, it is customary to initialise the convolution filters of each layer using random Gaussian matrices of sizes that are layer- and CNN-architecture-dependent. Here, we shall focus on the effect of surgery on 3 × 3 Gaussian matrices. To illustrate the effect of SVD surgery on point clouds of convolutions, we generate a set of $10^{4}$ 3 × 3 matrices drawn from the Gaussian distribution N(0,0.01). We use the norm of the original matrix, the norm of the inverse, and the condition number to illustrate the effects of SVD surgery and observe the distribution of these parameters per set. Figure 4 below shows a clear reduction in the condition numbers of modified matrices compared to the original ones. The reduction in the condition numbers is a result of reducing the norms of the inverses of the matrices (see Figure 5). The minimum and maximum condition numbers for the original set are approximately 1.2 and 10,256, respectively. After only replacing the smallest singular value $\sigma_{3}$ with $\sigma_{2}$, after reconstruction, the new minimum and maximum values are 1.006 and 17.14, respectively.  

Figure 4 shows a significant change in the distribution of the norms of the inverses of 3 × 3 matrices post-surgery, which is consequently reflected in their condition number distribution. The use of a linear combination formula helps keep the range of condition numbers below a certain threshold depending on the range of singular values. For instance, 3D illustrations in Figure 5 show a significant reduction in the condition number by keeping the ranges below 3 in (b) and 2 in (c), where $\sigma_{2}$ and $\sigma_{3}$ are replaced with $\sigma_{1}/3+2\sigma_{2}/3$ and $(\sigma_{1}+\sigma_{2})/2$, respectively. The new minimum and maximum condition number values for both sets after matrix surgery are [1.004,2.687] and [1.003,1.88], respectively.

### 5.3. Effects of SVD Surgery on PDs of Point Clouds of Matrices

For the motivating application, we need to study the impact of SVD surgery on point clouds of matrices (e.g., layered sets of convolution filters) rather than single matrices. Controlling the condition numbers of the layered point clouds of CNN filters (in addition to the fully connected layer weight matrices) during training affects the model’s learning and performance. The implementation of SVD surgery can be integrated into customised CNN models as a filter regulariser for the analysis of natural and US image datasets. It can be applied at filter initialisation when training from scratch, on pretrained filters during transfer learning, and on filters modified during training by backpropagation after every batch/epoch.

In this section, we investigate the topological behaviour of a set of matrices represented as a point cloud using persistent homology tools, as discussed in Section 4. For any size n×n filters, we first generate a set of random Gaussian matrices. By normalising their entries and flattening them, we obtain a point cloud in $S^{n\times n-1}$ residing on its (n×n−1)−sphere. Subsequently, we construct a second point cloud in $S^{n\times n-1}$ by computing the inverse matrices, normalising their entries, and flattening. Here, we only illustrate this process for a specific point cloud of 3×3 matrices for two different linear combinations of the two lower singular values. The general case of larger-size filters is discussed in the first author’s PhD thesis [46].

**Figure 6:**
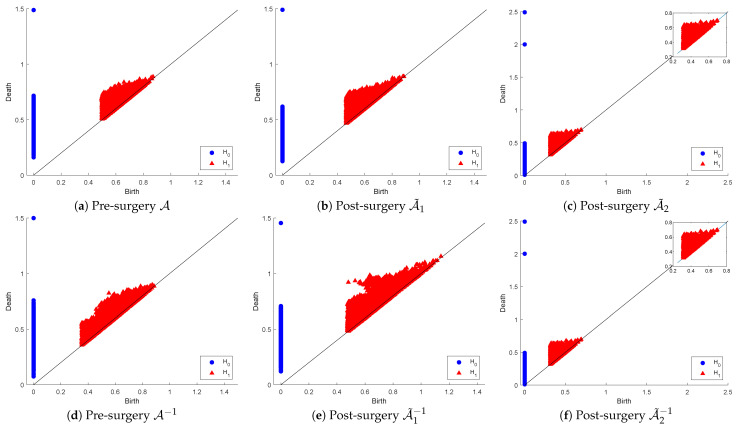
Persistence diagram of point clouds A and $A^{-1}$ before and after SVD-based surgery.

Figure 6 below shows the $H_{0}$ and $H_{1}$ persistence diagrams for point clouds (originals and inverses) plus those for post-matrix-surgery with respect to the linear combinations: (1) replacing both $\sigma_{2}$ and $\sigma_{3}$ with $\sigma_{1}$ (i.e., κ(A)=1) and (2) replacing $\sigma_{3}$ with $\sigma_{2}$. The first row corresponds to the effect of SVD on the PD of the original point cloud, while the second row corresponds to the inverse point cloud.

The original point cloud $\tilde{A}$ includes extremely wide-ranging matrices in relation to their conditioning, which means their proximity to the non-invertible set of matrices is also wide-ranging. That accounts for the observable visual differences between the PDs of $\tilde{A}$ and those of ${\tilde{A}}_{1}^{-1}$ in both dimensions. The PDs of ${\tilde{A}}_{1}$ and ${\tilde{A}}_{1}^{-1}$ are not significantly dissimilar in dimension 0, but in dimension 1, we can notice that many holes in ${\tilde{A}}_{1}^{-1}$ have longer lifespans, while many others are born later than the time that all holes in ${\tilde{A}}_{1}$ vanish. In fact, in dimension 0, the dissimilarities appear as a result of many connected components in ${\tilde{A}}_{1}^{-1}$ living longer than those in ${\tilde{A}}_{1}$. The PDs of ${\tilde{A}}_{2}$ and ${\tilde{A}}_{2}^{-1}$ are visually equivalent in both dimensions as a reflection of the fact that this surgery produces optimally well-conditioned orthonormal matrices (i.e., the inverse matrices are simply the transpose of the original ones). This means that the strict surgery that produces the ${\tilde{A}}_{2}$ point cloud is useful for applications that require orthogonality, whereas the less-relaxed surgery is beneficial for applications where condition numbers are in a reasonable range of values as long as they are not ill-conditioned.

For a more informative description of these observations, we computed the death-based binning table, which is shown below as Table 4. The results confirm that the topological profiles (represented by their PDs) of $\tilde{A}$ and ${\tilde{A}}_{1}^{-1}$ are indeed different in both dimensions. There is less quantitative similarity in dimension 0 between the PDs of ${\tilde{A}}_{1}$ and ${\tilde{A}}_{1}^{-1}$ than reported by visual examination. In dimension 1, the visual observations are to some extent supported by the number of holes in the various bins. The table also confirms the exact similarity in both dimensions of the PDs of ${\tilde{A}}_{2}$ and ${\tilde{A}}_{2}^{-1}$, as reported using visual examination.

Again, we estimated the proximities of the PDs of the various related pairs of point cloud matrices and their inverses in term of the bottleneck and the Wasserstein distance functions. The results are shown in Table 5 below. The significantly large distances in dimension 0 explain the noted differences between the PD of $\tilde{A}$ and that of ${\tilde{A}}^{-1}$. In dimension 1, the surprisingly small bottleneck distance between the PD of $\tilde{A}$ and that of ${\tilde{A}}_{1}^{-1}$ indicates that bottleneck distances may not reflect the dissimilarities in visual representations. The distances between the PDs of ${\tilde{A}}_{1}$ and ${\tilde{A}}_{1}^{-1}$ in both dimensions are reasonably small, except that in dimension 1, the distance increased slightly post the ${\tilde{A}}_{1}$ surgery. This may be explained by the observation made earlier that “many holes have longer lifespans, while many others are born later than the time that all holes in ${\tilde{A}}_{1}$ vanish” when visually examining the 1-dimensional PDs. Finally, these distance computations confirm the strict similarity reported above between the PDs of ${\tilde{A}}_{2}$ and ${\tilde{A}}_{2}^{-1}$.

#### SVD Surgery for the Motivating Application

The need for matrix surgery to improve the condition number arose during our previous investigation [46], which aimed to develop a CNN model for ultrasound breast tumour images that has reduced overfitting and is robust to reasonable noise. During model training, we observed that the condition numbers of a large number of the initialised convolution filters were fluctuating significantly over the different iterations [12]. Having experimented with various linear-combination-based SVD surgery techniques, the work eventually led to a modestly performing customised CNN model with reasonable robustness to tolerable data perturbations and generalisability to unseen data. This was achieved with a carefully selected constant linear combination SVD surgery applied to all convolutional layer filters at (1) initialisation from scratch, (2) pretrained filters, and (3) during training batches and/or epochs.

Our ongoing attempt to improve the previous work for improved CNN model performance is based on using more convolution layers and investigating the conditioning of the large non-square matrix of the fully connected layers (FCLs) of neurons. A major obstacle to the training aspects of this work is the selection of appropriate linear-combination-based SVD surgery for different point clouds for a larger range of filter sizes. In our motivating application as well as in many other tasks, it is specifically desirable to control the condition numbers of filters/matrices within a specific range and with reasonable upper bounds. Such requirements significantly increase the toughness of the challenge of finding different linear-combination-based surgery schemes (suitable for various convolutional layers and FCLs) that guarantee maintaining condition numbers within specified ranges.

There may exist many alternatives to using linear-combination-based reconditioning SVD surgery. The PH investigations of the last section indicate the need to avoid adopting crude/strong reconditioning algorithms to avoid slowing down learning and/or underfitting effects. Below is pseudocode, Algorithm 1, for a simple but efficient SVD surgery strategy that we developed more recently for “reconditioning” each of the convolution filters (as well as the components of the FCL weight matrices) after each training epoch that maintains the condition numbers within a desired range.
**Algorithm 1** SVD surgery and condition number threshold.**Input:** Filter *F* of size k×k, thresholding constant *C*1:Compute the SVD of *F*: $F=U\Sigma V^{T}$, and let $\left(\sigma_{1},\ldots ,\sigma_{k}\right)$ be the singular values of Σ in descending order.2:$x\leftarrow \sigma_{1}/C$, j←k                                    ▹ Initial threshold3:**while** $\left(\sigma_{j}>x\right)\wedge \left(j>1\right)$ **do**4:    $\sigma_{j}\leftarrow x$                             ▹ Threshold small singular values5:    j←j−16:    $x\leftarrow \sigma_{j}$                             ▹ Update threshold for next iteration7:**for** i=j to k−1 **do**                       ▹ Smoothen the remaining singular values8:    $\sigma_{i+1}\leftarrow \left(\sigma_{i+1}+\sigma_{i}\right)/2$9:Reconstruct *F* using the modified singular values: $F\leftarrow U\Sigma V^{T}$**Output:** Filter *F* of size k×k

Note that the above algorithm does not change any input matrix that has a condition number in the specified range, while it makes minimal essential adjustments to the singular values. We are incorporating this efficient SVD-based reconditioning procedure during the training of specially designed SLIM CNN models for tumour diagnosis from ultrasound images. The results are encouraging, and future publications will cover the implications of such “reconditioning” matrix surgery on the performance of Slim-CNN models and the topological profiles of the filters’ point clouds during training.

Future works include (1) assessment of topological profiles of point clouds of matrices (and those of their inverses) in terms of their condition number distribution and (2) quantifying Demmel’s assertion that links condition numbers of matrices to their proximity to non-invertible matrices. For such investigation, the SVD surgery scheme is instrumental in generating sufficiently large point clouds of matrices for any range of condition numbers.

## 6. Conclusions

We introduced simple SVD-based procedures for matrix surgery to reduce and control the condition number of an n×n matrix by conducting surgery on its singular values. Persistent homology analyses of point clouds of matrices and their inverses helped formulate a possible PD version of Demmel’s assertion. Recognising the challenge of using the convex linear combination strategy to stabilise the performance of CNN models, a new, simpler-to-implement matrix reconditioning surgery is presented.

## Figures and Tables

**Table 1:** The persistency binning of well-conditioned and ill-conditioned point cloud PDs.

Set	Bins	0–0.2	0.2–0.4	0.4–0.6	0.6–0.8	0.8–1	1–1.2	1.2–1.4	1.4–2.5
$X_{1}$	#C.C	0	2	7	9	22	12	9	3
	#Holes	0	0	0	0	0	1	14	55
$X_{1}^{-1}$	#C.C	0	3	5	13	24	14	4	1
	#Holes	0	0	0	0	1	0	10	45
$X_{2}$	#C.C	0	1	5	41	16	0	0	1
	#Holes	0	0	0	0	11	59	1	0
$X_{2}^{-1}$	#C.C	4	12	31	16	0	0	0	1
	#Holes	0	0	1	6	20	17	0	0

**Table 2:** Comparison of bottleneck and Wasserstein distances.

	Bottleneck		Wasserstein (q = 1)		Wasserstein (q = 2)	
**PD Pairs**	**Dim 0**	**Dim 1**	**Dim 0**	**Dim 1**	**Dim 0**	**Dim 1**
$X_{1}$ and $X_{2}$	0.5717	0.1892	13.45988	9.31917	1.98646	1.03191
$X_{1}^{-1}$ and $X_{2}^{-1}$	0.59606	0.24777	24.94575	13.0818	3.22486	1.37756
$X_{1}$ and $X_{1}^{-1}$	0.1385	0.1172	3.00973	2.50156	0.46242	0.39456
$X_{2}$ and $X_{2}^{-1}$	0.38591	0.22174	16.23648	4.44692	2.09447	0.61168

**Table 3:** Euclidean norms and condition numbers before and after matrix surgery.

Matrix	∥A∥	$∥A^{-1}∥$	κ(A)
*B*	0.041883482	2034.368572	85.20644044
${\tilde{B}}_{1}$	0.041883482	199.5721482	8.358776572
${\tilde{B}}_{2}$	0.041883482	30.36464182	1.271776943
${\tilde{B}}_{3}$	0.041883482	23.87576058	1

**Table 4:** The persistency binning of the various PDs before and after SVD surgery.

Set	Bins	0–0.1	0.1–0.2	0.2–0.3	0.3–0.4	0.4–0.5	0.5–0.6	0.6–0.7	0.7–2.5
$\tilde{A}$	#C.C	0	39	390	2606	5717	1208	39	1
	#Holes	0	0	0	0	195	5828	15314	1776
${\tilde{A}}^{-1}$	#C.C	33	637	3194	3290	2031	729	84	2
	#Holes	0	0	0	1091	5114	3839	3213	1374
$\tilde{A_{1}}$	#C.C	52	598	2946	5465	932	6	0	1
	#Holes	0	0	0	101	3456	8997	2702	136
${\tilde{A_{1}}}^{-1}$	#C.C	45	489	2379	4935	1942	207	2	1
	#Holes	0	0	0	29	1952	5994	2854	1672
$\tilde{A_{2}}$	#C.C	591	3119	5129	1135	24	0	0	2
	#Holes	0	0	0	962	2641	1703	107	0
${\tilde{A_{2}}}^{-1}$	#C.C	591	3119	5129	1135	24	0	0	2
	#Holes	0	0	0	962	2641	1703	107	0

**Table 5:** Comparison of bottleneck and Wasserstein distances.

	Bottleneck		Wasserstein (q = 1)		Wasserstein (q = 2)	
**PD Pairs**	**Dim 0**	**Dim 1**	**Dim 0**	**Dim 1**	**Dim 0**	**Dim 1**
$\tilde{A}$ and ${\tilde{A}}^{-1}$	500.000	0.100710	1566.893	911.103	707.169	6.732
${\tilde{A}}_{1}$ and ${\tilde{A}}_{1}^{-1}$	0.093	0.149820	222.803	319.057	2.570	3.509
${\tilde{A}}_{2}$ and ${\tilde{A}}_{2}^{-1}$	0	0	0	0	0	0

## Data Availability

The data presented in this study are available on request from the corresponding author.
